# Mineralogical imprints of earthquake activity in sedimentary structures

**DOI:** 10.1038/s41598-026-45025-y

**Published:** 2026-03-20

**Authors:** Szymon Świątek, Karolina Lewińska, Małgorzata Pisarska-Jamroży, Christina Günter

**Affiliations:** 1 Faculty of Geographical and Geological Sciences, Adam Mickiewicz University, Krygowskiego 12, Poznań, 61-680 Poland; 2 Institute of Geosciences, University of Potsdam, Karl-Liebknecht-Str. 24- 25, 14476 Potsdam, Germany

**Keywords:** liquefaction, seismite, mineral structures, earthquake, siderite, Environmental chemistry, Natural hazards, Sedimentology, Mineralogy

## Abstract

Seismic activity can leave distinct imprints in sediments; however, the geochemical and mineralogical conditions controlling sediment liquefaction and deformation remain incompletely understood. Here, we present a comprehensive study combining laboratory experiments and field samples to investigate the formation of seismically-induced structures, with a particular focus on sideritic and core-rim features. Core-rim structures (CRS) were consistently identified across all experimental and field conditions subjected to shocks, independent of the degree of water mineralization, the presence of iron compounds, or experimental duration. The observations suggest that their formation is largely controlled by physical processes during seismic events, indicating that CRS may represent a potentially indicator of seismic activity in unconsolidated sediments. Additionally, variations in their morphology and elemental distribution appear to reflect fluid flow pathways, providing insights into fluid redistribution during seismic deformation in the field. Sideritic structures, observed exclusively in field samples and laboratory variants with FeO(OH), displayed ring-like morphologies and were primarily composed of FeO and carbonate minerals. Multivariate statistical analyses, including cluster and principal component analysis (PCA), revealed strong similarities between these environments, pointingto comparable geochemical conditions during their formation. Overall, this studydemonstrates the value of integrating experimental and field-based approachesto improve our understanding of seismic deformation processes. Core-rim and sideritic structuresmay contribute useful evidencefor interpreting past seismic events and for refining criteriaused to identify seismites and reconstruct fluid migration patterns in sedimentary systems.

**Supplementary Information:**

The online version contains supplementary material available at 10.1038/s41598-026-45025-y.

## Introduction

Earthquakes may leave behind distinct geological evidence both near and far from their epicenters^1^. This evidence ranges from surface fissures and faults, landslides, and turbidites to seismically-induced, deformed sedimentary layers known as seismites^2–5^. Seismites form due to the reduction in shear resistance in water-saturated clastic sediments during seismic waves, triggering liquefaction and fluidization processes that develop a variety of soft-sediment deformation structures (SSDS)^1,4,6,7^​​.

Seismically-induced liquefaction, a process where sediments lose cohesion and behave temporarily as a liquid or plastic mass, is key in understanding seismically-induced SSDS^6,8^. This state occurs due to increased pore pressure which mobilizes grains, often resulting in features such as load casts, pseudonodules, clastic volcanoes, water-escape structures, or dish-and-pillar structures^6–18^. However, a significant challenge in sedimentology and paleoseismology is differentiating between SSDS triggered by seismic activity and those formed by other trigger mechanisms, such as storms, volcanoes or rapid aggradation rate^5,8,18^.

Recent studies have begun to determine the unique micro-sized evidence left by seismic events^5,19^. Notably, the formation of gold within quartz grains has been observed, which also provides a direct indicator of seismic events^20,21^. Despite these advances, no comprehensive geochemical and mineralogical analysis has yet focused on SSDS, not only to provide a clear distinction between seismogenic and non-seismogenic features​ but also to check which minerals are found in liquefied sediments.

This study aims to bridge this knowledge gap by presenting the first experimental investigation into the mineralogical records associated with SSDS. Our objectives include identifying the post-seismic mineralogical alterations in unconsolidated sediments, exploring the geochemical conditions that influence these records, and establishing a methodology for recognizing seismogenic SSDS based on mineralogical criteria.

## Materials and methods

### Materials and experimental setup

Materials and methods are connected to these proposed by Świątek et al.^21^. The study utilized unconsolidated siliciclastic sediments (silt and fine-grained sand) collected from natural outcrops in NW Poland (52°55’54.8"N 15°46’52.8"E and 52°51’52.0"N 15°58’00.9"E), and classified following Friedman and Sanders^22^. Sediments were saturated with distilled, medium-, and highly-mineralized water, with iron compounds added to replicate natural conditions^23^.

Sediment samples were placed in 108 transparent plexiglass cylinders in layers, with iron (II) sulfate (54 samples) or iron oxyhydroxide solutions (54 samples; after^24,25^. Samples were incubated under reducing conditions and analyzed at intervals of 0, 1.5, 3, 6, 9, and 12 months. At each time point, seismic shock simulations were conducted using an Analysette 3 Spartan shaking table (magnitude 3.5, 50 Hz, 3 mm amplitude, 15 s). The classification as a magnitude 3.5 event in laboratory context refers not to direct energy equivalence, but to acceleration characteristics (e.g., peak ground acceleration) and scaling relationships that simulate the mechanical impact of such earthquakes at smaller spatial and temporal scales. Detailed description of these seismic vibrations was explained in Świątek and Pisarska-Jamroży^26^. Samples were prepared with great precision and care to ensure consistency across all experimental conditions. The experimental setup was specifically designed to eliminate the possibility of SSDS formation due to non-seismic factors such as loading or rapid sedimentation. Developed seismites were analyzed by micromorphological analyses. The detailed scheme of experimental setup was illustrated in Fig. S1.

### Source of iron compounds

**Fig. 1:**
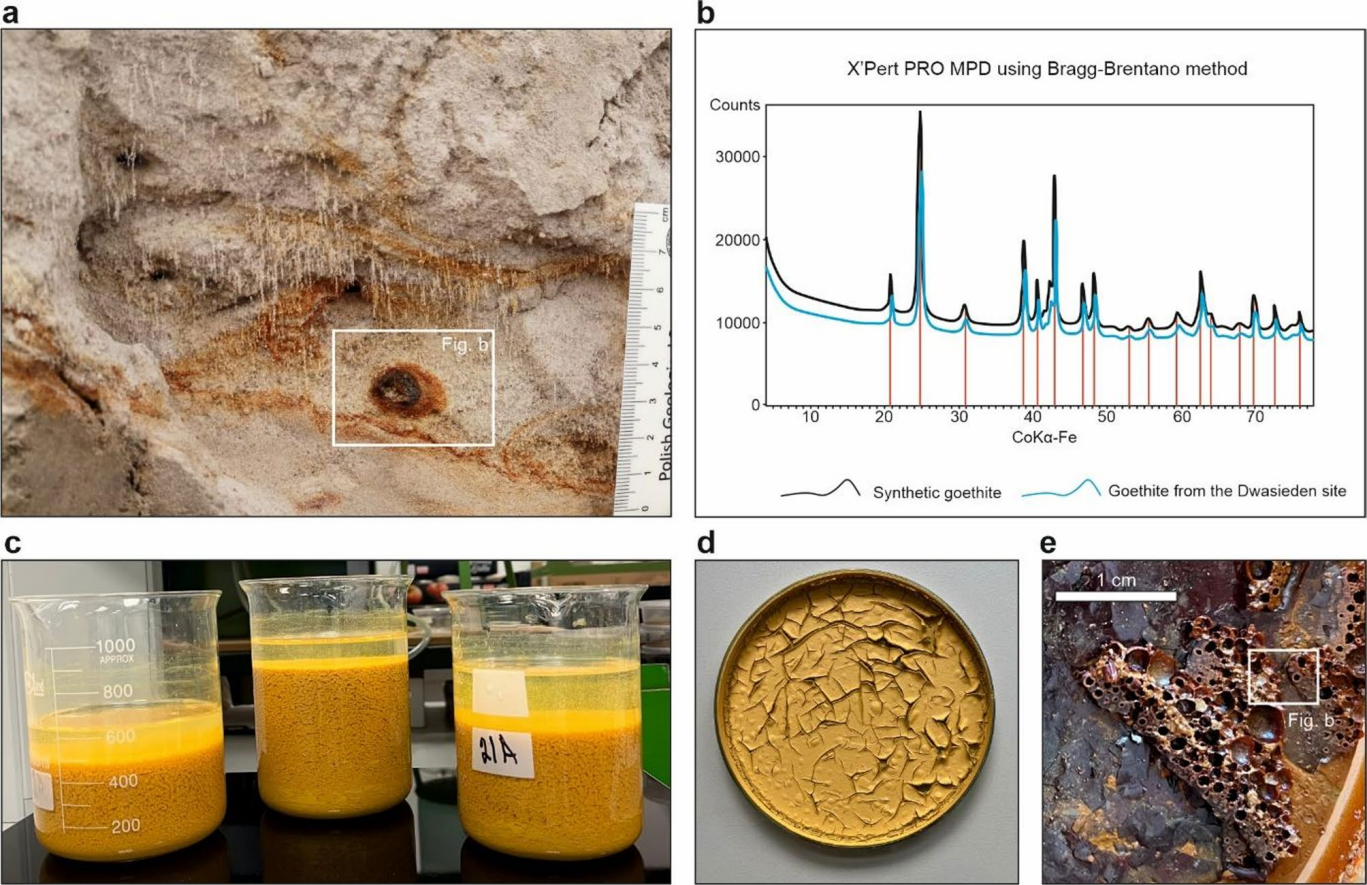
Stages of research preparation. (**a**) Goethite accumulations observed at the Dwasieden field site. (**b**) XRF analysis of iron-rich material conducted at the University of Warsaw, confirming goethite identification in samples from Dwasieden and obtained in the laboratory. (**c**) Preparation of synthetic goethite, including heating and neutralization to pH 7. (**d**) Dried synthetic goethite. (**e**) Crystallization of goethite within laboratory samples.

Based on field observations we studied iron accumulations there (Fig. 1a) and analyzed using Raman spectroscopy (Fig. 1b).

To conduct these experiments, in turn, two types of iron compounds were used: iron (II) sulphate and iron (III) oxide-hydroxide, marked as goethite (Fig. 1b). First of them, i.e., iron (II) sulphate, as an easily soluble salt, was commercially purchased from WarChem (Poland). Second, later called goethite, was designed to simulate the naturally occurring goethite in soils and which is more difficult to dissolve in the environment and commonly form in suboxic to mildly oxidizing conditions in near-surface sediments, such as shallow marine or glaciofluvial systems. These settings often show episodic redox shifts and active iron cycling^27^. In laboratory conditions, FeO(OH) was synthesized following the method of Böhm^24^, after being modified by Schwertmann and Cornell^25^. The procedure involved preparing goethite by mixing 1 M Fe(NO_3_)_3_ and 5 M KOH, dissolving them in distilled water, and heating the solution at 70 °C for 72 h. The final step involved neutralizing the resulting precipitate to pH 7 by washing and centrifugation (Fig. 1c). The synthesized goethite (Fig. 1d) was then mixed with the commercially obtained iron (III) oxide-hydroxide (from Linegal Chemicals company) in a 1:3 ratio. Initially, a layer of sand was positioned at the cylinder bottom and saturated with an iron (II) sulphate solution. This solution, i.e., 7 g of iron per 250 ml of water, aimed to imitate a natural iron concentration of approximately 0.5-1%^23^. After gravitational processes, the next layer was added and so on. The cylinders were then incubated in low-light, closed conditions to minimize evaporation and promote reducing conditions. The same approach was applied to iron oxyhydroxide (Fig. 1d-e).

### Micromorphological analyses

The samples (after seismic shocks) were subsequently air-dried at room temperature, and approximately 100 g of undisturbed sediment were collected and placed into a PVC tube. These prepared samples were immersed in Araldite 2020 epoxy resin for several days to solidify the sediment structure. Thin sections were later prepared and analyzed with a Scanning Electron Microscope (SEM).

Micromorphological analyses of unconsolidated sediments were conducted using carbon-coated thin sections at the Faculty of Geographical and Geological Sciences (Adam Mickiewicz University in Poznań, Poland), the Faculty of Geology (University of Warsaw, Poland), and at the University of Potsdam (Germany). The analysis involved a detailed examination of thin sections, with 5–7 random SEM images taken at magnifications ranging from 10 to 500 μm. These images were used to assess the micromorphological features of the sediments, particularly focusing on new mineral objects and structures, developed after the seismically-induced liquefaction phenomenon.

In addition, new and post-seismic mineralogical evidence were assessed for other features, such as the presence, and the relationships of chemical compounds. To achieve this, the elemental composition of specific areas on the grain surface was determined using energy-dispersive X-ray spectroscopy (EDX) analysis, and the distribution of elements was visualized through EDX mapping using a ZEISS Sigma VP SEM and JEOL JSM-6510 coupled with an XPlore EDX-detector from Oxford Instruments. The final analyses also considered the geochemical conditions of the sediments where the unconsolidated sediments were located.

### Reference material

Morphological analyses were also performed on the same sand that had not been exposed to seismic shocks (Fig. S2), as well as on sediment samples taken directly from a well-documented earthquake-deformed outcrop at the Dwasieden site in Germany (54°30’04.2"N 13°36’56.7"E; Fig. 1a). This site, dated to the Late Pleistocene, features two seismites containing various SSDS, including pseudonodules, load casts, flame structures, fluid-escape structures, and ball-and-pillow structures^15,27^. The detailed description of this site, including its tectonic setting, is provided by Pisarska-Jamroży et al.^15^ and Pisarska-Jamroży et al. (2018)^28^. In addition, to compare, samples were also collected from the Sārnate outcrop in western Latvia (57°04′01.34"N, 21°24′55.88"E), where SSDS such as load casts, injection structures, and small-scale faults have been well documented^29^. These structures, in turn, are interpreted as the result of storm-induced loading processes^29,30^(Fig. S2). Both sites havewell-documented SSDS^31^.

### Statistical analyses

Based on the EDX mapping analysis, the elemental composition (expressed as oxide wt%) of the studied mineral structures was used to create scatter plots, linear correlation analyses using Pearson’s coefficient, and trend lines between the examined variables. Additionally, multivariate analyses were conducted, including triangular plots, dendrogram analyses, principal component analysis (PCA), and box-and-whisker plots. Statistical significance tests were also performed, such as the Kruskal-Wallis H test, to compare the data for statistically significant differences. These methods provided a comprehensive approach to understanding the relationships and variability within the mineralogical data.

## Results and discussion

### Seismite-associated deformation and mineral structures

**Fig. 2:**
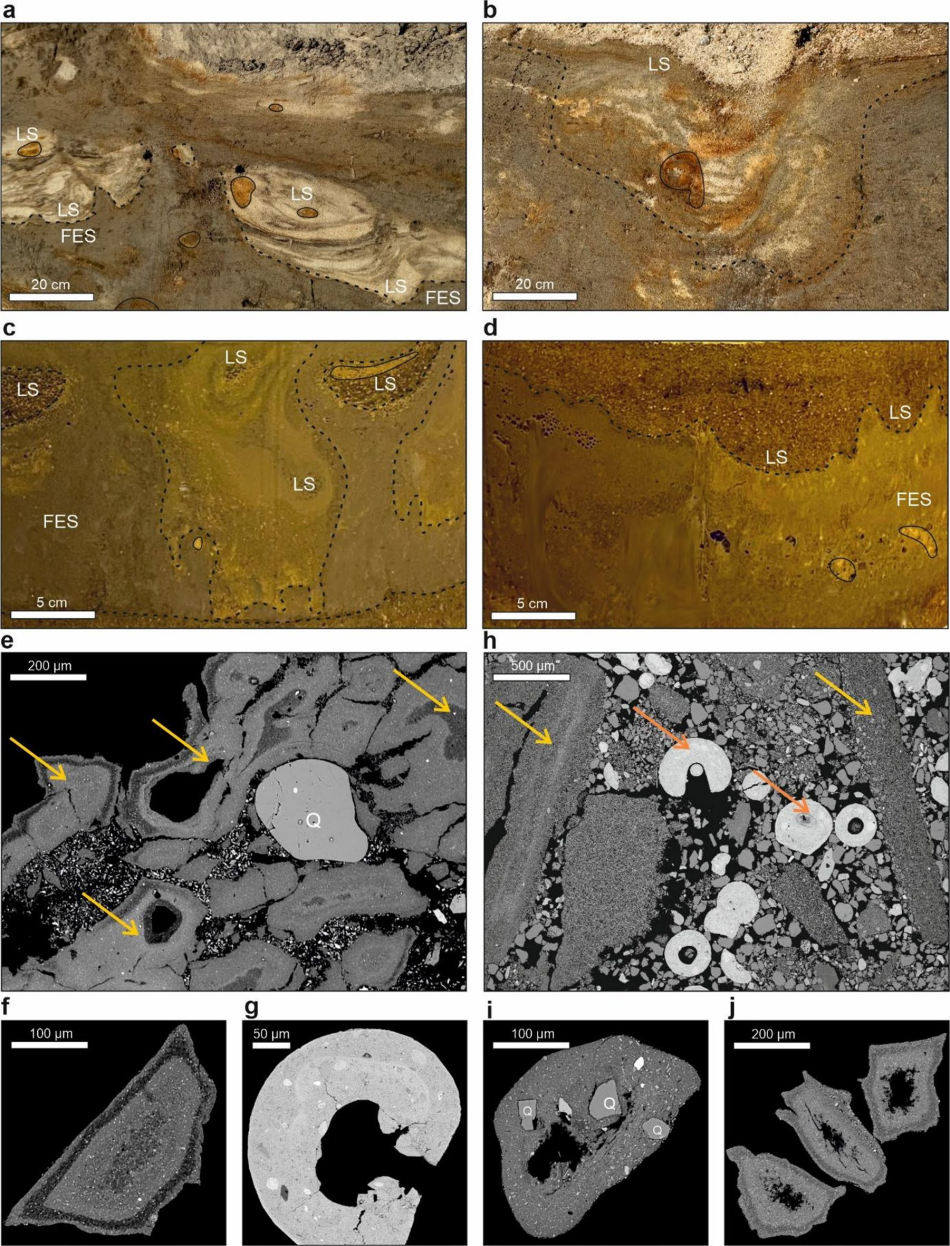
Seismite-associated structures recognized at the Dwasieden field site and developed in laboratory conditions. (**a**,**b**) The Dwasieden site. (**a**) Co-occurrence fluid-escape structures (FES) with load structures (LS) within goethite accumulation. (**b**) Intra-layered LS with iron-rich compounds. (**c**,**d**) Laboratory conditions. (**c**) Iron-rich FES and LS with goethite accumulation. (**d**) Co-occurrence FES with goethite accumulation and LS. The black dashed line indicates the structure boundary. The continuous black line indicates the zone of goethite accumulation. (**e**–**j**) New seismically-induced mineral structures recognized at the field site and laboratory experiments. (**e**–**g**) The Dwasieden site. (**e**) Core-rim structures (yellow arrows) surrounding sand-sized fracturing quartz grain. (**f**) Single CRS with visible zonation. (**g**) Siderite structure (orange arrows). (**h**–**j**) Laboratory conditions. (**h**) CRS co-occurring with siderite structures and sand-sized quartz grains. (**i**) CRS containing quartz and calcite grains. (**j**) Multiple CRS.

Samples from both laboratory experiment variants and the field site at Dwasieden (NE Germany), as well as at Sārnate site were collected and analyzed (Figs. 1a and 2).The Dwasieden study site has been characterized by two seismites containing fluid-escape structures (FES) and load structures (LS) (Fig. 2a,b). Additionally, we sampled rusty accumulations within these structures, which were identified as goethite, and confirmed with XRF analysis (Fig. 1a,b). The FES were characterized by a high content of silt and clay fractions, with a relatively low proportion of sand fraction^15,28,32^. In contrast, the LS were dominated by a sand fraction, with a minor presence of finer sediments. Within these structures, layers with visible iron compounds could be observed (Fig. 1b). The Sārnate site, located in western Latvia, exposes rhythmically laminated sandy-silty deposits with SSDS such as LS, FES, and small-scale faults (Fig. S2). The sediments are dominated by the fine sand fraction, with subordinate amounts of silt, and are moderately to well sorted. Micromorphological samples were taken from the deformed layers, but no visible iron accumulations were observed there. Laboratory experiments, in turn, led to the development of similar seismite-associated SSDS to those observed in the field (Fig. 2c). They exhibited visible internal lamination enriched with primary iron compounds, along with orange-rusty goethite accumulations (Fig. 2c-d). These SSDS formed the basis for further detailed mineralogical, geochemical, and microstructural analyses, allowing to investigate the formation processes and potential geological conditions that could facilitate their development both in natural and controlled laboratory settings.

Detailed micromorphological analysis of both laboratory and field samples revealed mineral structures whose presence regardless of the experimental variant, degree of water mineralization, or the type of iron compounds used (FeO(OH) and FeSO_4_). In Dwasieden site we observed a specific structures, which was called by us as a core-rim structures (CRS). They consist of a central, usually empty core, encased by a distinct rim with a contrasting zonation (Fig. 2e–j). Their consistent appearance across all conditions highlights their robustness and suggests that their formation is driven by fundamental physical processes rather than specific geochemical variations. In contrast, no comparable CRS were identified in the samples from the Sārnate site. Given the absence of such features (Fig. S2e), distinctly observed in both the laboratory variants and the Dwasieden reference site, the Sārnate material was excluded from further detailed analyses presented in the subsequent sections of this study. We believe that CRS can be an indicator, with their potential as a novel criterion for identifying seismic activity in both experimental and natural sedimentary environments.

At the Dwasieden site, as well as in all samples with FeO(OH) additions, we identified the presence of siderite (Fig. 2g,h), confirmed by SEM-EDX as well as Raman apparatus (Fig. S3). These formations exhibited distinct ring-like morphologies, sometimes appearing as semi-closed structures and, less frequently, as fully closed (Figs. 1a and 2).

### Structures indicating fluid-escape direction

The FES (Fig. 2) may be key for understanding the dynamics of seismically-induced liquefaction within sedimentary environments and almost always are indirect indicators of seismic events^5,6,10,33^. During the liquefaction process, various structures develop, which serve as evidence of fluid flow direction (Fig. 2; cf^34–36^. Among the most significant of these structures are clastic volcanoes, injection structures, clastic dykes, and clastic sills^6,10,33,37^.

**Fig. 3:**
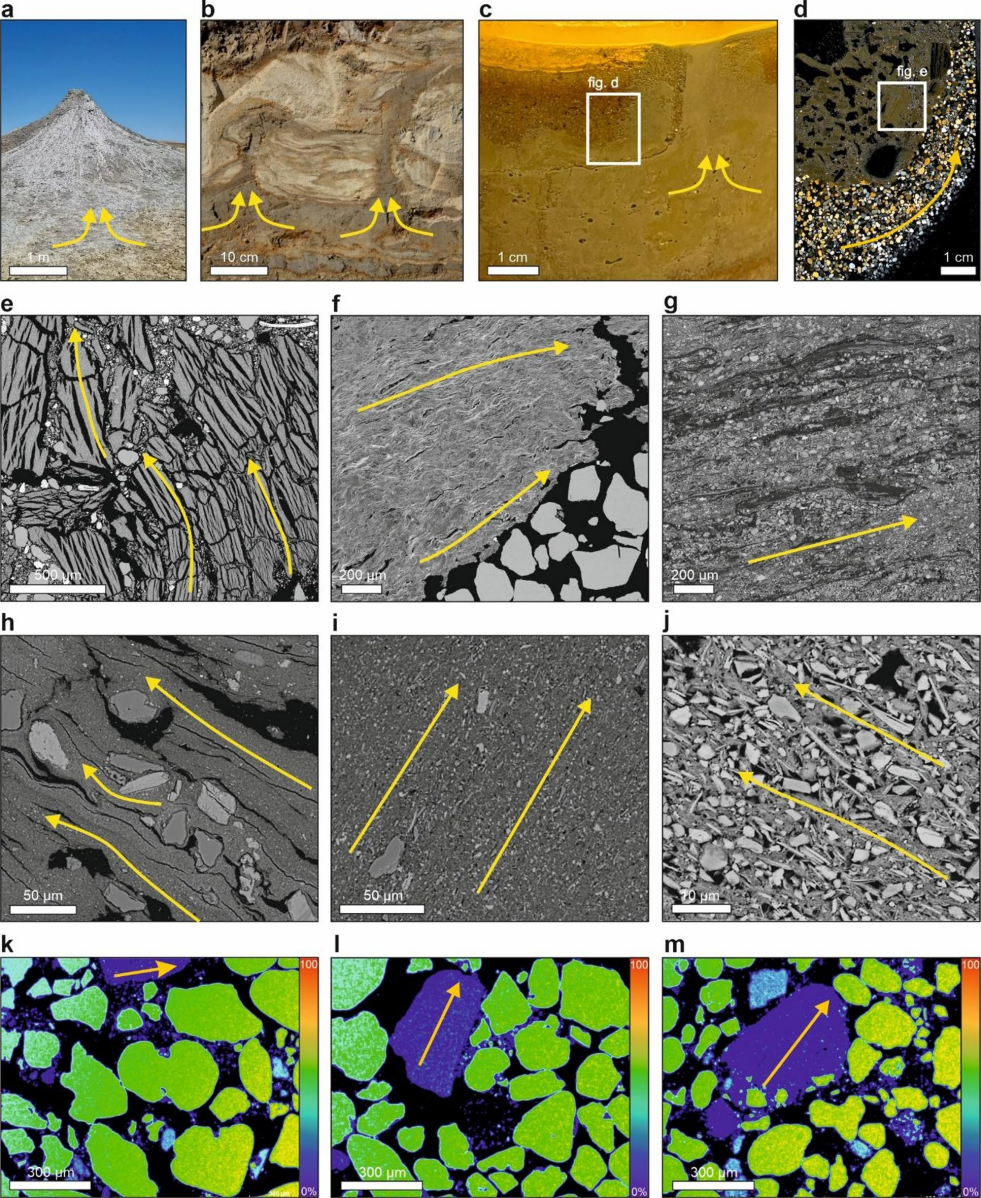
Seismite-associated structures, as well as CRS in varying scales. (**a**) Mud volcano in Azerbaijan. (**b**) FES recognized at the Dwasieden study site. (**c**) FES developed in this study under laboratory conditions. (**d**) Scan of thin section from laboratory sample. (**e**–**k**) CRS in wide-range magnification collected from differential laboratory samples. (**e**) Disturbed CRS. (**f**–**j**) CRS parallel to quartz and other mineral grains. (**k**–**m**) CRS-associated distribution of silica. Yellow arrows indicate flow direction.

Understanding the direction of fluid-escape during seismogenic liquefaction is essential for reconstructing past seismic events and the processes influencing sediment deformation (cf^36^. Determining these pathways provides insight into the intensity and dynamics of seismic forces and their effects on sediment behavior and deposition. Additionally, identifying fluid escape directions allows for a better understanding of how sediment properties, seismic forces and geological conditions influence styles of sediment deformation (cf^38,39^. Fluid-escape structures linked to seismogenic liquefaction have varied widely in scale, ranging from several meters to mere centimeters (Figs. 2 and 3). The CRS also provide new insights into fluid movement and escape during sediment liquefaction. The core likely may be the primary fluid path, while the rim forms from peripheral flow and escape processes, providing a clear indication of the directional flow of fluids (Fig. 3). The presence of CRS in both controlled laboratory conditions and natural settings suggests that these formations are fundamental to fluid-escape processes.

Core-rim structures appeared as massive, plastic-like masses with a textureless, where no individual crystals, minerals, or grains were visible in the matrix (Figs. 2e,f,j and 3e–g). Occasionally, these structures displayed foliation, indicating some material alignment despite the absence of discernible grain boundaries (Fig. 3f). This textureless matrix suggests rapid formation under intense fluid pressure or high shear stress during seismic events, conditions that inhibit grain preservation^40^. In addition to the textureless matrix, CRS exhibited a grain-in-matrix texture, with a dominant groundmass interspersed with regularly spaced grains of quartz, feldspar, calcite, and rutile (Figs. 2e and 3h,i). This texture mimics the appearance of isolated minerals in an amorphous groundmass, resembling igneous rock features but resulting here from intense pressure and deformation rather than slow cooling^41^. Under high magnification, some areas of CRS displayed a patterned texture, with mineral grains showing regularity and alignment (Fig. 3j). These regions suggest conditions of reduced pressure or mineral alignment during the post-liquefaction phase (cf^21,42^. The elemental distribution within CRS, including their incorporation into specific minerals, further supports their role as indicators of fluid flow direction and the dynamics of seismically-induced deformation (Fig. 3k–m).

**Fig. 4:**
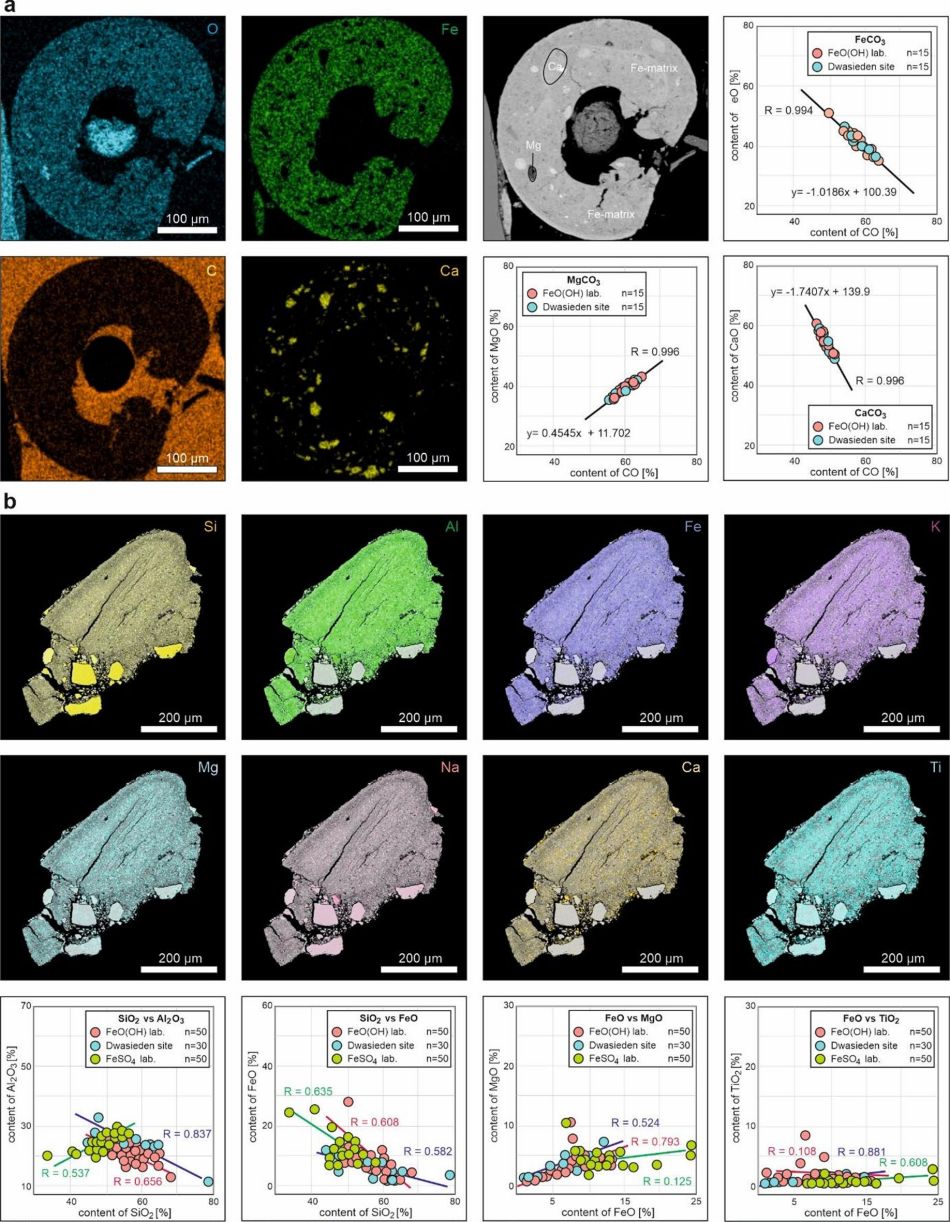
EDS-mapping of analyzed structures. (**a**) Siderite structure with scatterplots of the main elements. (**b**) CRS with distribution of main elements accumulation, as well as scatterplots of the main elements and relationships.

Sideritic structures provide valuable insights into fluid-escape pathways, as their formation is closely linked to geochemical conditions that govern fluid migration in sediments. Their iron-rich composition and zonation patterns can serve as indicators of paleo-fluid flow, capturing evidence of past fluid movements within unconsolidated sediments. The presence of these structures suggests localized chemical reactions influenced by redox conditions, which can help reconstruct fluid migration dynamics during seismic or diagenetic processes (Fig. 4a).

These observationsoffer insights into the dynamic processespotentially involved in the formation of CRS, suggestingthat mineral reorganization may occur under seismic pressure.Similar processeshave been proposed in previous studies^43,44^. The observed textural variability, ranging from textureless to grain-in-matrix and patterned forms, may reflect interactions between fluid dynamics, mineral plasticity, and shear stress. Such features could be related to fluid-escape pathways and mineral compounds transformationsassociated with seismic deformation. The CRScontribute to the understanding of FES by potentially recording pathways of fluid migration. Theirdistinctive configuration and compositional similaritiessuggest that they may serve asuseful indicators for reconstructing seismic fluid-escape pathways, with possible implications forassessing fluidization-related effects on sediment propertiesin different geological settings.

### Implications of (paleo)geochemical conditions

We conducted detailed chemical analyses on both sideritic and core-rim structures (Fig. 4). These analyses allowed us to investigate the geochemical composition and stability of these features, providing insight into the (paleo)environmental conditions under which they formed.

**Fig. 5:**
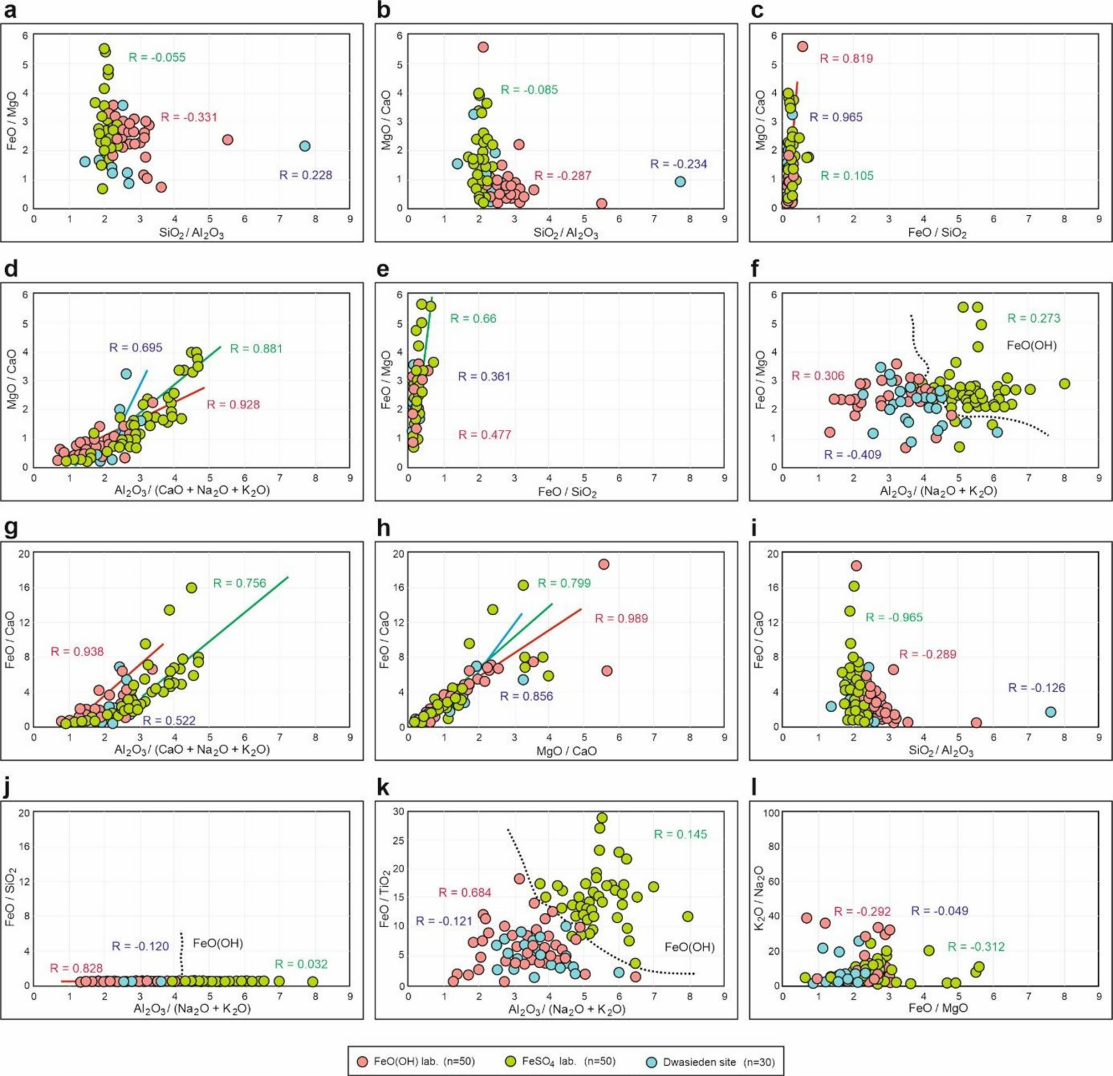
Scatter plots of selected oxide ratios with coefficient of Pearson correlation. For correlations with *R* > 0.65, trend lines were generated. Some relationships are observed only within specific variants, while others display trends consistent across all laboratory and field samples. Certain plots clearly differentiate the values of the FeO(OH) variant from the others.

The sideritic structures appear to be a stable, probably irreversible form consisting of an iron-rich matrix with embedded clasts of calcium (Ca) and magnesium (Mg) carbonates (Figs. 2g and 4a). The base of the structure is primarily composed of FeO and carbon compounds, creating a dense and hardy matrix. In some areas, bright spots can be observed, which are composed predominantly of CaO. Dark, sporadically distributed clasts within the structure are formed by MgO compounds. We also performed a comparative analysis between sideritic structures synthesized in the laboratory (FeO(OH)) and those collected from the Dwasieden site (Fig. 4a). Correlation coefficient analysis showed no significant differences in the elemental composition across those two groups, suggesting that the experimental conditions effectively replicate natural processes (Fig. 5, Fig. S4). Moreover, the combined analysis of field and laboratory samples yielded a statistically significant high Pearson’s coefficient, approaching *R* ~ 1 (Table S1). This high correlation indicates that the sideritic structures are geochemically consistent, regardless of their origin and experimental variants. Their development and preservation highlight the importance of iron and carbonate availability in sedimentary systems, providing valuable information about the geochemical environment during seismically-induced liquefaction. Siderite typically forms in reducing environments where oxygen levels are low^45,46^. These conditions are common in water-saturated sediments, such as swamps, peat bogs, and marine or lacustrine sediments with limited oxygen diffusion^45^.

The CRS do not exhibit a diverse distribution or concentration of elements, suggesting that any observed internal zoning within the structures is not a result of chemical processes but rather of physical factors, most likely pressure and compression (Fig. 4b). These formations are primarily composed of silicon (Si), aluminum (Al), iron (Fe), potassium (K), magnesium (Mg), sodium (Na), calcium (Ca), and titanium (Ti). The chemical composition and oxide proportions closely resemble those found in pseudotachylite, a well-documented seismic indicator occurring in vein form within fault and subduction zones^47–49^. However, pseudotachylite, a product of high-pressure and high-temperature conditions associated with seismic activity, is traditionally thought to form exclusively in igneous or metamorphic rock environments^47–49^. The compositional consistency across various samples suggests that, like pseudotachylite, CRS could potentially capture a record of physical forces without the need for conditions typically required in magmatic environments^49,50^. This insight broadens the understanding of seismic deformation processes, suggesting that CRS may develop under a wider range of geological conditions.

**Fig. 6:**
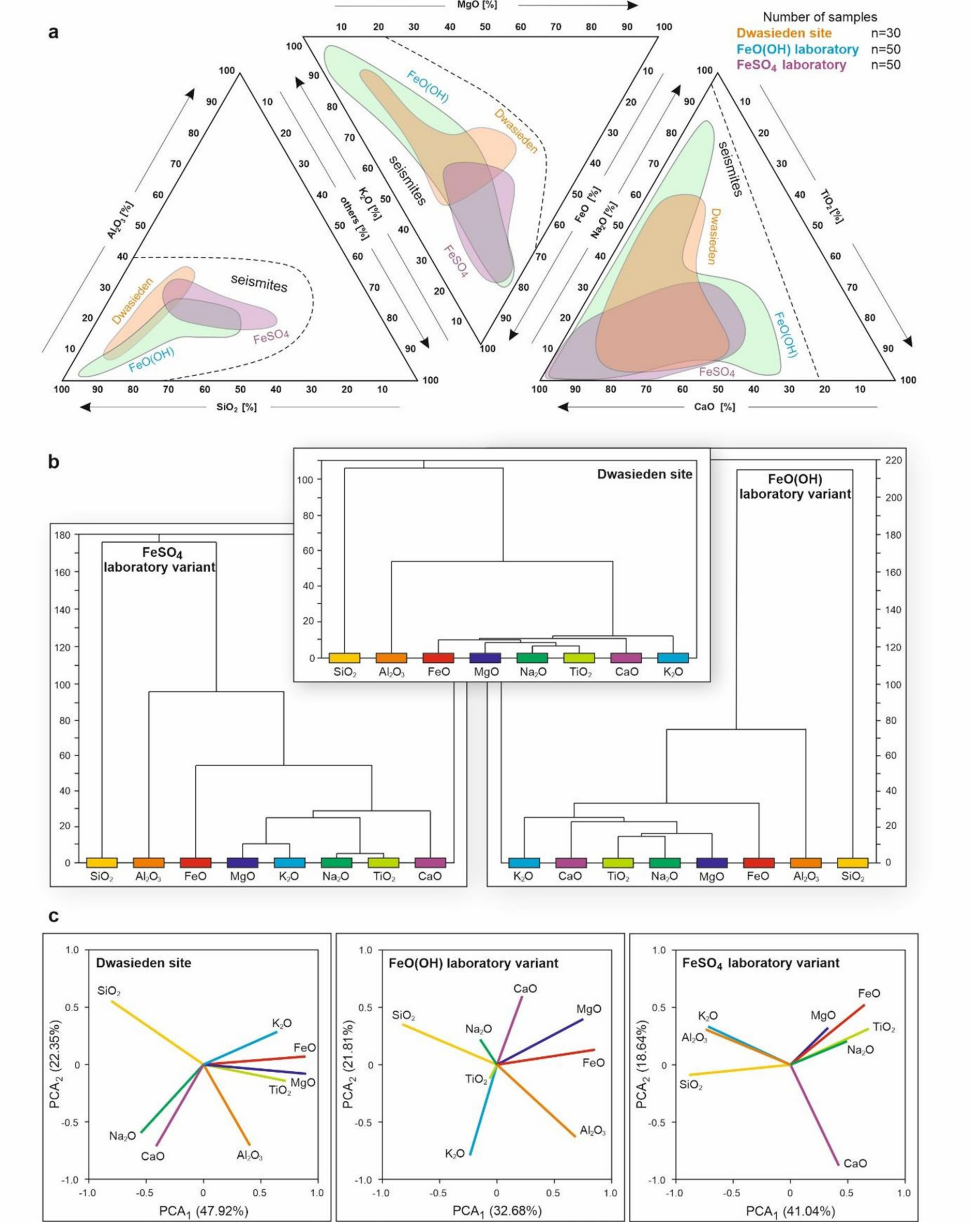
Statistical multivariate graphs for laboratory variants (FeO(OH), FeSO_4_) and Dwasieden samples including CRS. (**a**) Triangular plots showing relations between chemical components to each other. Each triangle indicates separation in 100%. The following chemical compounds in every triangle were transformed. (**b**) Dendrogram connections for all analyzed components. (**c**) Principal Component Analysis (PCA) for each analyzed variant. All samples exhibit a consistent trend, with FeO(OH) and Dwasieden samples showing the greatest similarity in their shared characteristics.

We also conducted three statistical analyses, examining the composition and proportions of various chemical compounds within CRS from two laboratory variants and field samples. Relationships between the primary components in these structures reveal notable similarities between the laboratory variant with FeO(OH) and the field samples, although the FeSO₄ variant does not differ significantly (Fig. 6, Fig. S4). Both FeO(OH) and the Dwasieden site samples exhibit a comparable trend in the concentrations of Al and Si relative to other components, and similar levels of Mg, K, Fe, as well as Na, Ca, and Ti (Figs. 4b and 6a).

Cluster analysis also confirmed the close resemblance between the field samples and those obtained in the laboratory using goethite (FeO(OH)). It is worth highlighting that in all three variants, the strongest associations were observed between sodium oxide and titanium, followed by magnesium and potassium (Fig. 6b). The roles of iron, aluminum, and silicon appear to be secondary in the formation of CRS and may depend on other local factors. As a third analytical approach, we performed PCA, which further substantiated the similarity between the FeO(OH) samples and those from Dwasieden site, particularly in the comparable behavior of Fe and Mg, as well as Na and Ti in the FeSO₄ variant (Fig. 6c). These analyses indicate that the compositional trends in FeO(OH) and Dwasieden samples are closely aligned, while also showing that FeSO₄ samples share significant similarities in specific elemental distributions. The combined statistical evidence from PCA and cluster analysis suggests that FeO(OH)-related CRS and Dwasieden samples likely formed under similar geochemical conditions, reflecting parallel fluid migration dynamics and mineral structures deposition processes (Figs. 2e and j, 4b, 5 and 6).

## Conclusions

This study presents the first large scale of laboratory experiments (108 samples) designed to explore how geochemical conditions may influence sediment liquefaction and the development of associated deformation and mineral structures. Based on the results, the following conclusions can be proposed:

1. Core-rim structures (CRS) were consistently identified across all analyzed samples, irrespective of applied chemical conditions. This consistency suggests that CRS formation is largely controlled by physical processes associated with seismic loading, rather than specific geochemical variations. Consequently, CRS may represent a common feature of seismically deformed unconsolidated sediments and potentially useful indicator of seismic activity. Core-rim structures were not observed in sediment samples deformed by non-seismic processes, supporting their potential specificity to seismic loading conditions.
2. Sideritic structures were observed exclusively in samples from the Dwasieden site and in laboratory variants containing FeO(OH), suggesting that their formation is likely linked to particular geochemical conditions, including reducing environments. Their presence in both natural and experimental settings suggests that they may serve as markers of paleoseismic events where such conditions are met.
3. The morphology and elemental composition of CRS provide insights into fluid migration during seismic liquefaction. The internal structure of CRS likely reflects fluid flow pathways, with the core representing the primary fluid escape route and the rim forming due to peripheral flow processes. These structures thus enhance the ability to reconstruct past seismic fluid dynamics in sedimentary environments.
4. Multivariate statistical analyses, including cluster analysis and principal component analysis (PCA), revealed strong similarities between CRS from FeO(OH) laboratory variants and those from the Dwasieden site. These similarities suggest that comparable geochemical and physical conditions may have influenced CRS formation in both settings, supporting their potential relevance for interpreting seismic deformation and associated fluid migration.
5. The experimental approach reproduced deformation features comparable to those observed in natural sediments, demonstrating itsusefulness for investigating process related to seismic liquefaction. Overall, this studycontributes to refining the criteriaused to identify seismites andimproves our understanding of the role of fluid flow in sedimentary deformation processes

## Supplementary Information

Below is the link to the electronic supplementary material.

Supplementary Material 1

Supplementary Material 2

## Data Availability

All data to reproduction this results are available at [https://doi.org/10.5281/zenodo.14902032](https:/doi.org/10.5281/zenodo.14902032).
