# Supplementary material for: Mineralogical imprints of earthquake activity in sedimentary structures
Source: Sci Rep. 2026 Mar 20;16:14307. doi: 10.1038/s41598-026-45025-y (PMC13144493; doi:10.1038/s41598-026-45025-y)
Supplement: Supplementary file 1 — Supplementary Material 1 [file 41598_2026_45025_MOESM1_ESM.docx]

**SUPPLEMENTARY TABLES**

| Dwasieden | SiO_2_ | Al_2_O_3_ | FeO | MgO | CaO | Na_2_O | K_2_O | TiO_2_ |
| --- | --- | --- | --- | --- | --- | --- | --- | --- |
| SiO_2_ | 1 | -0.9 | -0.53 | -0.74 | -0.37 | -0.68 | -0.76 | -0.66 |
| Al_2_O_3_ | -0.9 | 1 | 0.46 | 0.73 | 0.21 | 0.74 | 0.53 | 0.52 |
| FeO | -0.53 | 0.46 | 1 | 0.62 | 0.45 | 0.35 | 0.21 | 0.41 |
| MgO | -0.74 | 0.73 | 0.62 | 1 | 0.47 | 0.6 | 0.34 | 0.45 |
| CaO | -0.37 | 0.21 | 0.45 | 0.47 | 1 | 0.19 | 0.34 | 0.43 |
| Na_2_O | -0.68 | 0.74 | 0.35 | 0.6 | 0.19 | 1 | 0.31 | 0.42 |
| K_2_O | -0.76 | 0.53 | 0.21 | 0.34 | 0.34 | 0.31 | 1 | 0.37 |
| TiO_2_ | -0.66 | 0.52 | 0.41 | 0.45 | 0.43 | 0.42 | 0.37 | 1 |

| FeO(OH) | SiO_2_ | Al_2_O_3_ | FeO | MgO | CaO | Na_2_O | K_2_O | TiO_2_ |
| --- | --- | --- | --- | --- | --- | --- | --- | --- |
| SiO_2_ | 1 | -0.88 | -0.38 | -0.61 | -0.31 | -0.45 | -0.25 | -0.33 |
| Al_2_O_3_ | -0.88 | 1 | 0.37 | 0.63 | 0.2 | 0.48 | 0.33 | 0.3 |
| FeO | -0.38 | 0.37 | 1 | 0.36 | 0.08 | 0.16 | -0.03 | -0.1 |
| MgO | -0.61 | 0.63 | 0.36 | 1 | 0.34 | 0.38 | 0.15 | 0.07 |
| CaO | -0.31 | 0.2 | 0.08 | 0.34 | 1 | 0.16 | -0.08 | 0 |
| Na_2_O | -0.45 | 0.48 | 0.16 | 0.38 | 0.16 | 1 | -0.01 | 0.01 |
| K_2_O | -0.25 | 0.33 | -0.03 | 0.15 | -0.08 | -0.01 | 1 | 0.06 |
| TiO_2_ | -0.33 | 0.3 | -0.1 | 0.07 | 0 | 0.01 | 0.06 | 1 |

| FeSO_4_ | SiO_2_ | Al_2_O_3_ | FeO | MgO | CaO | Na_2_O | K_2_O | TiO_2_ |
| --- | --- | --- | --- | --- | --- | --- | --- | --- |
| SiO_2_ | 1 | 0.73 | 0.62 | 0.25 | -0.86 | 0.4 | 0.84 | -0.51 |
| Al_2_O_3_ | 0.73 | 1 | 0.85 | 0.72 | -0.96 | 0.02 | 0.89 | 0.14 |
| FeO | 0.62 | 0.85 | 1 | 0.67 | -0.91 | 0.44 | 0.66 | -0.01 |
| MgO | 0.25 | 0.72 | 0.67 | 1 | -0.64 | -0.22 | 0.62 | 0.56 |
| CaO | -0.86 | -0.96 | -0.91 | -0.64 | 1 | -0.27 | -0.9 | 0.09 |
| Na_2_O | 0.4 | 0.02 | 0.44 | -0.22 | -0.27 | 1 | -0.03 | -0.77 |
| K_2_O | 0.84 | 0.89 | 0.66 | 0.62 | -0.9 | -0.03 | 1 | 0.03 |
| TiO_2_ | -0.51 | 0.14 | -0.01 | 0.56 | 0.09 | -0.77 | 0.03 | 1 |

**Table S1. Pearson correlation coefficients between the analyzed variables for each variant.** This table provides insight into the strength and direction of linear relationships across laboratory and field conditions.
