# Supplementary material for: Mineralogical imprints of earthquake activity in sedimentary structures
Source: Sci Rep. 2026 Mar 20;16:14307. doi: 10.1038/s41598-026-45025-y (PMC13144493; doi:10.1038/s41598-026-45025-y)
Supplement: Supplementary file 2 — Supplementary Material 2 [file 41598_2026_45025_MOESM2_ESM.docx]

**Supplementary Materials**

The supplementary material contains Figure S1 representing experimental design. Figure S2 illustrates SEM-image natural sediment and that subjected to seismic shocks, as well as SEM-image for storm and loading-induced soft-sediment deformation structures. Figures S3 presents Raman spectra confirming the presence of siderite. Figure S4 presents box-and-whisker plots. Table S1 indicates Pearson correlation coefficients between the analyzed variables for each variant.

**
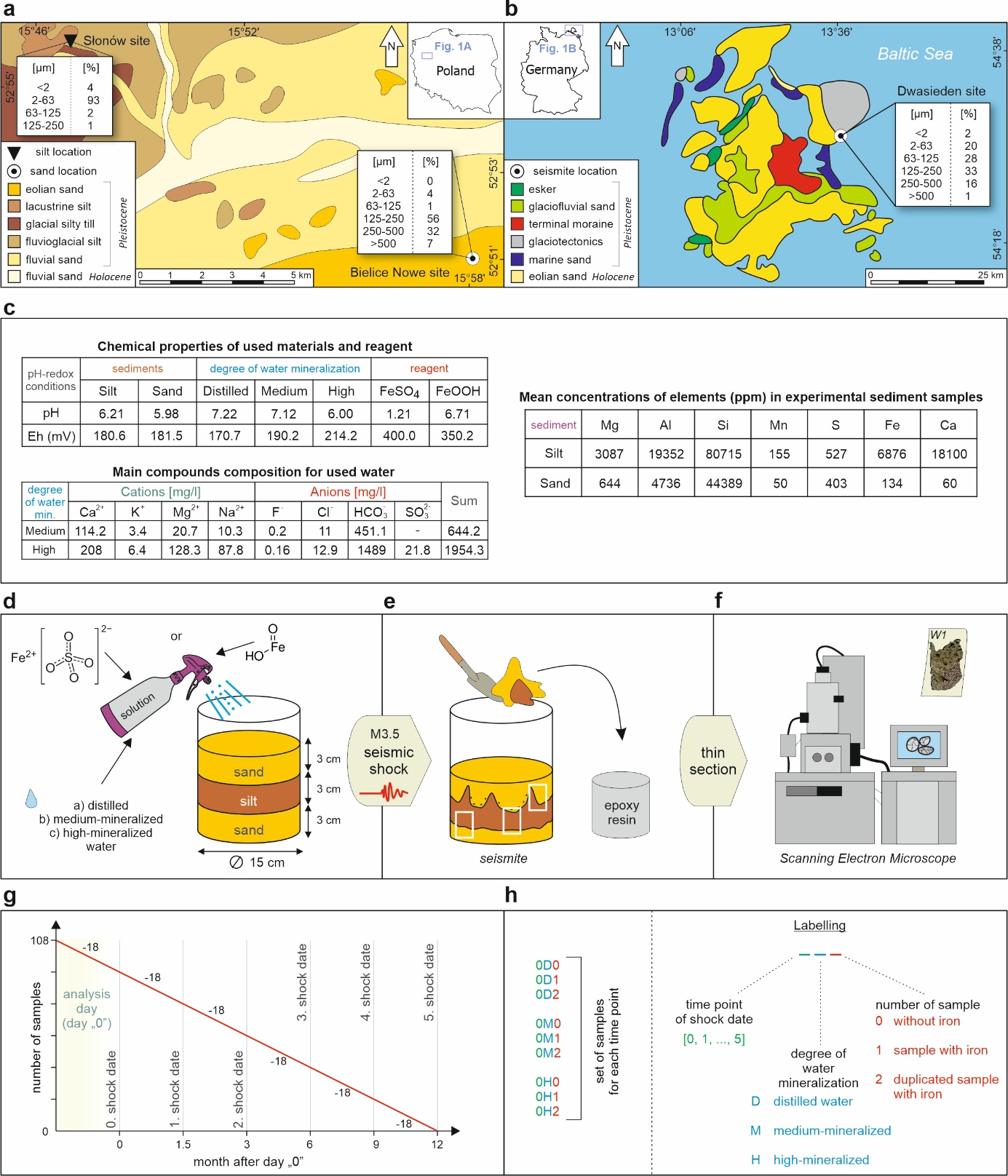
**

**Figure S1. Experiment design (after Świątek et al., 2025; modified).** **(a)** Location and simplified lithogenetic map of the Słonów and Bielice Nowe sites in the northwest Poland (Świątek et al., 2025). **(b)** Simplified geological map of Rügen island with the studied Dwasieden site (The map was created using CorelDRAW Graphics Suite 21.0 (https://www.coreldraw.com/pl/product/coreldraw/). **(c)** Chemical properties of used materials and chemical composition of water. **d-f**. Workflow of preparation and technical conditions of samples. **(g)** Time chart of experiment duration. **(h)** Set of samples for each analysis day, with the labelling.

**
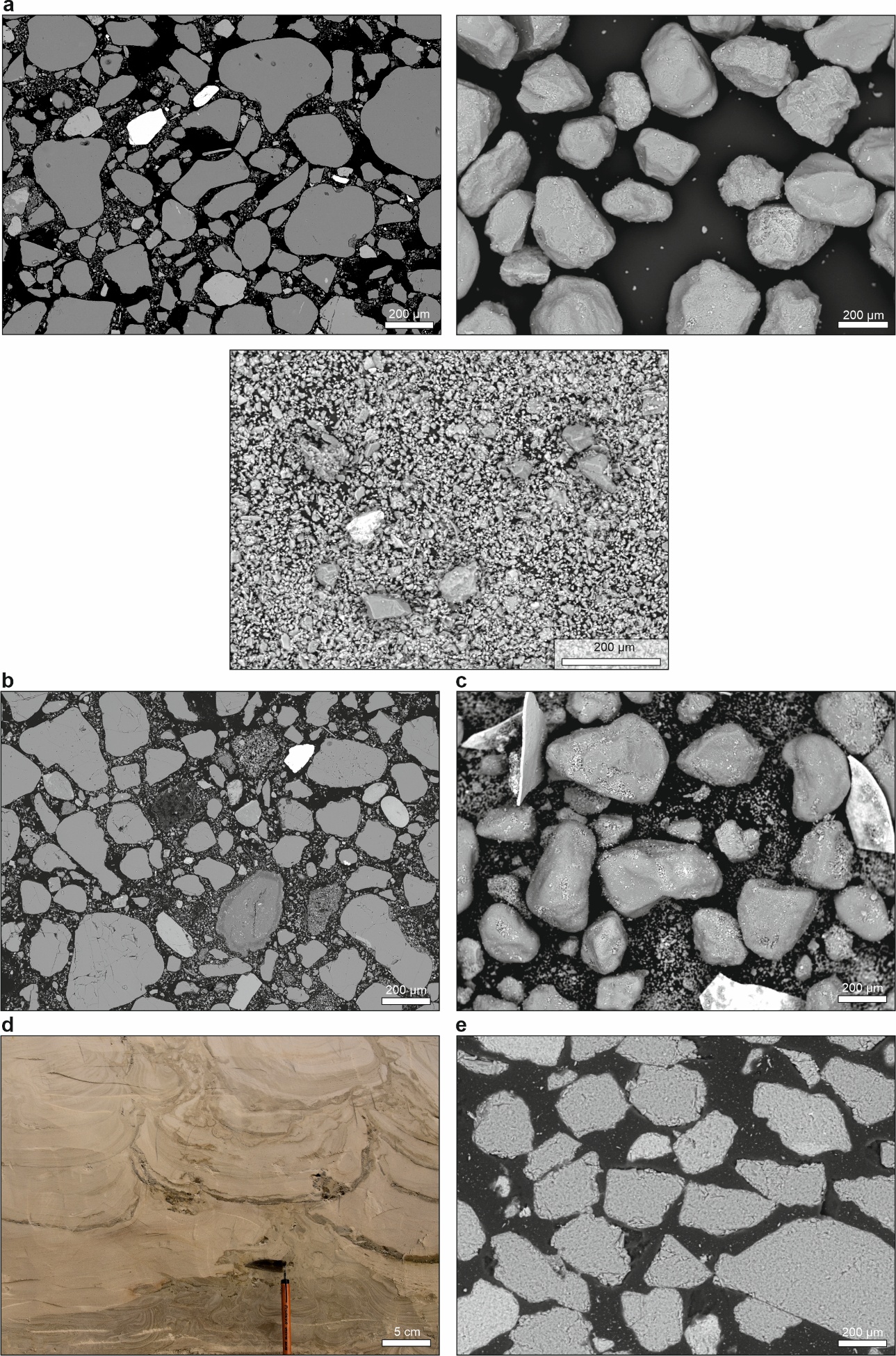
**

**Figure S2.** **Reference material for this study.** **(a).** Sediments with minerals do not subject to simulated seismic shocks (from the Bielice Nowe site). **(b)** Sediments with minerals recognized in the seismite samples from the Dwasieden site. **(c)** Sediments with minerals recognized in undeformed samples from the Dwasieden site. **(d)** Storm- and loading-induced soft-sediment deformations structures recognized at the Sārnate site. **(e)** Textural features of deformed sediments at the Sārnate site.


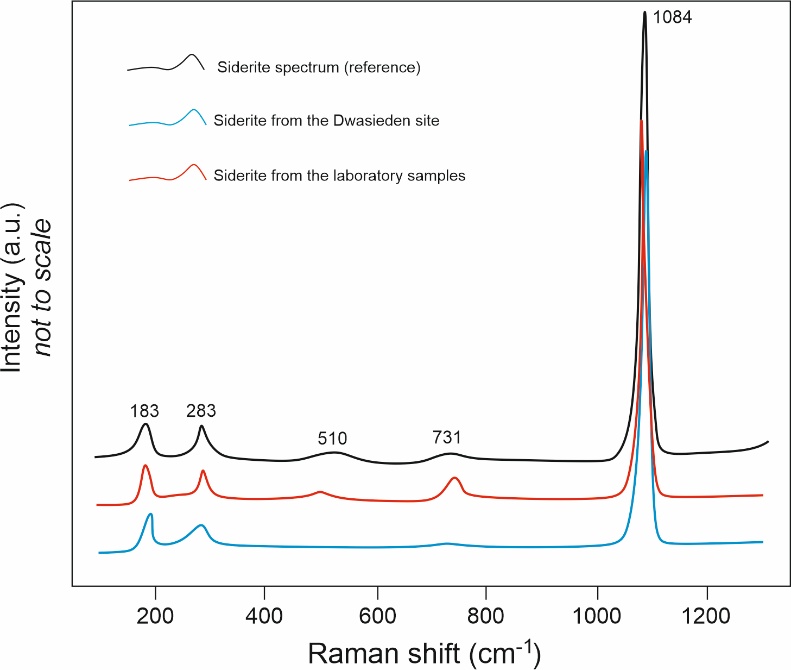


**Figure S3.** Raman spectra confirming the presence of siderite (FeCO₃) identified in both laboratory-formed and field-collected samples. Characteristic peaks at ~1085 cm⁻¹ (CO₃²⁻ symmetric stretch), ~285 cm⁻¹ and ~740 cm⁻¹ are consistent with reference siderite spectra, supporting its formation under reducing conditions in both experimental and natural settings.

**
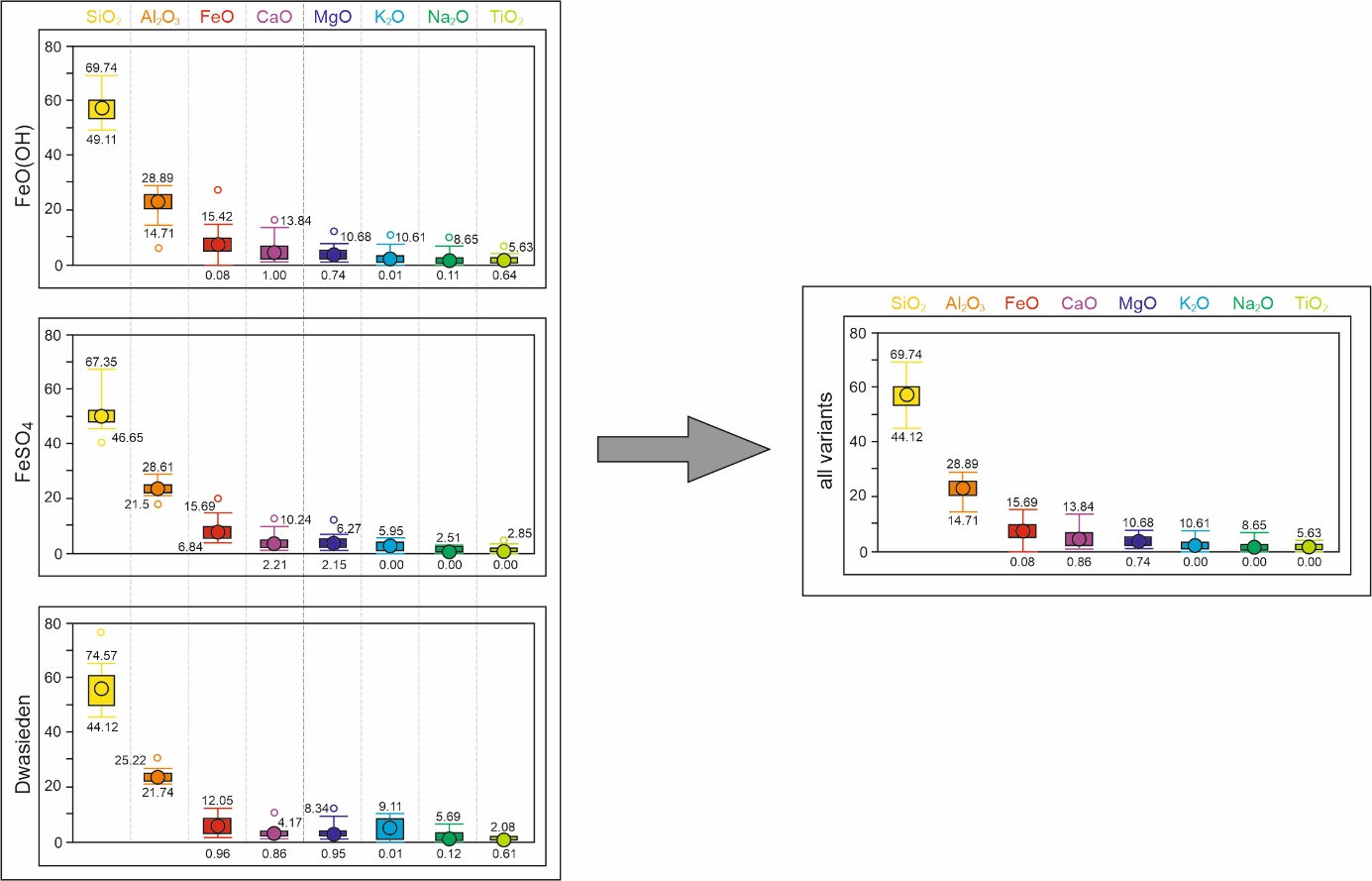
**

**Figure S4. Box-and-whisker plots for each variant separately and combined.** The largest amplitudes are observed in the FeO(OH) variant, with values tightly concentrated between the 25^th^ and 75^th^ percentiles. The Dwasieden site variant exhibits a wide range of values for individual oxides. Circles indicate outer values.
